# The influence of inorganic components and carbon-oxygen surface functionalities in activated hydrothermally carbonized waste materials for water treatment

**DOI:** 10.1007/s11356-020-09839-1

**Published:** 2020-07-03

**Authors:** Mirva Niinipuu, Kenneth G. Latham, Stina Jansson

**Affiliations:** 1 Department of Chemistry, Umeå University, Linnaeus väg 6, 90736, SE-90187 Umeå, Sweden; 2 Industrial Doctoral School, Umeå University, SE-90187 Umeå, Sweden

**Keywords:** Hydrochar, Biochar, Chemical activation, Sewage sludge, Horse manure, Linear regression analysis

## Abstract

In this study, we have examined how the activation of hydrothermally carbonized sewage sludge and horse manure influences the inorganic component of these materials and surface chemistry. This was examined through statistical correlations between kinetic tests using trimethoprim, fluconazole, perfluorooctanoic acid, and copper, zinc, and arsenic and physicochemical properties. Yield and inorganic content varied considerably, with potassium hydroxide–activated materials producing lower yields with higher inorganic content. Phosphoric acid activation incorporated inorganically bound phosphorus into the material, although this showed no statistically relevant benefit. A maximum surface area of 1363 m^2^g^−1^ and 343 m^2^g^−1^ was achieved for the horse manure and sewage sludge. Statistical analysis found positive correlations between carbon-oxygen functionalities and trimethoprim, fluconazole, perfluorooctanoic acid, and copper removal, while inorganic content was negatively correlated. Conversely, arsenic removal was positively correlated with inorganic content. This research provides insight into the interactions with the organic/inorganic fraction of activated waste materials for water treatment.

**Electronic supplementary material:**

The online version of this article (10.1007/s11356-020-09839-1) contains supplementary material, which is available to authorized users.

## Introduction

The conversion of waste materials into useful value-added products is a prerequisite for achieving a sustainable society. Furthermore, the conversion method must also be environmentally benign, with the final product aimed at replacing a non-sustainable product. Activated carbon is an ideal candidate for replacement, as it is commonly produced from non-renewable fossil-based resources, such as peat, lignite, and coal (Marsh and Rodríguez-Reinoso 2006a). Activated carbon can also be produced from a wide range of bio-resources, such as coconut husk; however, these may be better utilized in the production of chemicals from emerging bio-refineries (Lee and Lavoie 2013).

Activated carbon is currently employed as an adsorbent material in wastewater treatment facilities for the removal of organic micropollutants, such as pharmaceuticals, personal care products, and perfluorinated compounds (Cooney 1998; Hansen et al. 2010; Wong et al. 2018). These facilities also produce waste products in the form of sludges (Saveyn et al. 2005), which can be converted into the activated carbon that can be fed back into the water treatment cycle. Sewage sludge also contains a range of inorganic materials, such as iron, silicon, aluminum, phosphorus, potassium, and calcium compounds. These inorganic compounds may enhance the adsorption capacity of activated carbon produced from sewage sludge or other wet waste materials (Batzias and Sidiras 2004; Liu et al. 2012; Park et al. 2015; Shawabkeh 2006). Thus, sewage sludge is an ideal candidate for conversion into activated carbon, but an understanding of how the inorganic/organic fractions change during conversion into activated carbon is needed to optimize the process.

To produce activated carbon, the carbonaceous precursor needs to be heated to 450–1000 °C in the presence of an activation agent (e.g., steam, CO_2_, KOH, H_3_PO_4_, ZnCl_2_, Na_2_S_2_O_3_, C_2_K_2_O_4_) (Fuertes et al. 2018, Marsh and Rodríguez-Reinoso 2006b, c, Sevilla et al. 2017). These temperatures drive chemical reactions that remove carbon from the precursor or insert elements into the carbon matrix, creating pores that ultimately increase the surface area. The high surface area of activated carbon, up to 4000 m^2^g^−1^ (Cox and Mokaya 2017), provides activated carbon with a large adsorption capacity making it ideal for the removal of contaminants in waste water treatment (Wu and Tseng 2008). Unfortunately, the direct activation of sludge is not economically feasible due to its high water content (70–90%) (Saveyn et al. 2005). Thus, effective activation of sludge requires it to be initially dried or dewatered, representing a significant energy cost.

Hydrothermal carbonization (HTC), a wet, low temperature carbonization technique, has gained significant interest in the field of waste carbonization for its ability to efficiently process and dewater wet sludge materials (Danso-Boateng et al. 2015; Gao et al. 2019; Makela et al. 2016; Wang et al. 2019; Wang et al. 2017). HTC utilizes the auto-ionization of water to initiate a series of reactions, which break the C–O and C–C bonds in the precursor. This deconstructs the organic structure of the sludge, reducing its ability to retain water and releasing water bound in the cellular structure of bacteria contained in the sludge (Gao et al. 2019). The result of HTC is a solid carbonaceous material (hydrochar) that is easier to dewater than the sludge (Gao et al. 2019), an organic-rich supernatant and a small amount of gas.

HTC followed by activation of the solid hydrochar has been performed on a wide range of materials from saccharides to lignocellulosic biomass. Surface areas up to of 3420 m^2^g^−1^ have been achieved using KOH activation (Fuertes and Sevilla 2015), while H_3_PO_4_ activation has achieved surface areas of 2275 m^2^g^−1^ (Latham and Donne 2018). However, the HTC of sewage sludge to produce activated carbon has seldom been investigated (Khoshbouy et al. 2019; Kirschhofer et al. 2016) compared with using the products of HTC for energy use (Chen et al. 2019; Danso-Boateng et al. 2015; He et al. 2013; Merzari et al. 2019; Saha et al. 2019). Considering the potential to recycle sewage sludge into activated carbon at water treatment facilities, there is considerable scope to examine this area.

In this study, we have examined the adsorption capacity and physicochemical properties of a range of chemically activated (KOH and H_3_PO_4_), hydrothermally carbonized sewage sludge and horse manure. Horse manure was selected as a lignocellulosic-rich waste material to provide a comparison material to the sewage sludge.

The adsorption properties were studied via kinetic tests using trimethoprim, fluconazole, PFOA (perfluorooctanoic acid), Cu, Zn, and As as adsorbates in landfill leachate matrix. To further examine the inorganic/organic properties of the activated carbon materials, statistical correlations were calculated between the chemical composition of the material and the adsorbates to provide insight into adsorption interactions.

## Materials and methods

### Preparation of hydrochars

Anaerobically digested sewage sludge (SS) was sampled from a municipal wastewater treatment plant (Umeå, Sweden). A schematic overview of the wastewater treatment process in this plant was described previously by Ostman et al. (2018). Horse manure (HM) was collected in a riding school in Vännäs, Sweden, and it contains in addition to the fecal material also substantial amounts of sawdust and small wood chips that are used as bedding material. Each material was homogenized and then mixed separately with water until covered to form a slurry with a dry material content of approximately 5%.

Hydrothermal carbonization was carried out by adding 600 mL of slurry to a 1-L steel HTC reactor (Amar Equipments Pvt. Ltd.) and heated to either 180, 220, or 260 °C for 4 h. After 4 h, the reactor was cooled by an internal water-cooling system. The hydrochars were filtered and rinsed with deionized water and dried at 105 °C overnight in an oven.

The activated carbons were prepared by weighing 0.500 g of hydrochar and adding 2 or 4 g of KOH or 2 or 4 mL of H_3_PO_4_ to generate mixing ratios of 1:4 and 1:8 (weight ratio for hydrochar:KOH and weight:volume ratio for hydrochar: H_3_PO_4_), respectively. When KOH was used, 5 mL of deionized water was added in order to dissolve the KOH and wet the hydrochars. The mixture was dried in an oven at 105 °C overnight before being transferred into a furnace. The furnace was heated to 150 °C (heating rate 180 °C h^−1^) and kept there for 6 h to remove water from the H_3_PO_4_ samples before being ramped to 600 °C (at the heating rate of 360 °C h^−1^) and kept there for 1 h under nitrogen atmosphere. Temperature profiles are shown in SI in Fig. S1. After cooling, the activated materials were washed thoroughly with deionized water to remove the activation agents. The activated hydrochars were dried at 105 °C overnight and the yield of the resulting dry chars was calculated based on the dry matter content. The activated samples are denoted as raw material-HTC temperature-activation chemical-concentration, i.e., 220 °C sewage sludge hydrochar activated with KOH at 1:4 is SS-220-K-4. Control samples are denoted with control and the feedstock as RAW.

### Physical characterization

Scanning electron microscopy (SEM, Carl Zeiss Evo) was operated in low vacuum mode to study the surface morphology of the materials. The images were taken with a backscattered electron detector. Additionally, the elemental composition of the studied samples was studied with EDS (energy-dispersive X-ray spectroscopy).

The Brunauer-Emmett-Teller (BET) surface area was determined with a Micromeritics TriStar 3000 gas adsorption analyzer. Prior to analysis, samples of 50–100 mg were degassed for 2 h at 120 °C, under a continuous nitrogen flow, using a Micromeritics Smart Prep degassing unit. The specific surface areas were obtained by applying the BET to multi-point N_2_(g) adsorption/desorption isotherms. Micropore and mesopore volumes were determined using *t*-plot and Barrett-Joyer-Halenda (BJH) methods, respectively.

### Chemical characterization

Fourier transform infrared (FTIR) spectra were collected using a Vertex 70v spectrometer (Bruker Co., Germany). Samples were pressed onto an attenuated total reflectance (ATR) cell (single bounce diamond; Golden Gate, Specab) using a sapphire anvil. Absorption spectra were recorded at room temperature between 600 and 4000 cm^−1^, (resolution 2 cm^−1^, 100 co-added scans).

XPS analysis was performed with a monochromatic Al Kα source operated at 120 W. The spectra were collected with a Kratos Axis Ultra DLD electron spectrometer and processed with Kratos software. An analyzer pass energy of 160 eV and a pass energy of 20 eV were used for acquiring survey spectra and individual photoelectron lines, respectively. Spectrometer charge neutralization system was used to stabilize the surface potential. The binding energy (BE) scale was referenced to the C 1s line of aliphatic carbon, which was set to 285.0 eV. Using a Ni spatula, powder samples for the analysis were gently pressed into a pellet affixed to a sample holder. The limit of detection (LOD) was ~ 0.1 at. %.

### Adsorption tests

Landfill leachate was selected for the adsorption test matrix. It was sampled and frozen in plastic bottles directly after sampling and stored at − 18 °C. Prior to adsorption testing, the leachate was thawed and filtered through stacked glass wool, Whatman GB/B prefilter and GF/F filter (0.7 μm). The leachate was spiked with stock solutions containing trimethoprim, fluconazole, and PFOA to achieve final concentrations of 800 ng L^−1^. The leachate was also spiked with zinc(II) (TraceCERT®), copper(II) (TraceCERT®), and arsenic(V) (TraceCERT®) obtaining the final concentrations of 500 μg L^−1^ for Cu/Zn and 20 μg L^−1^ for As. The spiked concentrations were based on the concentrations found in wastewater plant influent in Sweden (Ostman et al. 2017). The characteristics of the filtered and spiked leachate are shown in Table S1 in the supplimentary information (analyzed by Eurofins AB, Lidköping).

The adsorption of contaminants was studied on six activated materials from hydrochar carbonized at 220 °C and two control samples. This series contained HM activated with KOH and H_3_PO_4_ at both ratios, and SS activated with KOH and H_3_PO_4_ at 1:4. The control samples were heat-treated HM-220 and SS-220, as well as commercial granulated activated carbon (GAC).

Kinetic adsorption tests were performed in 50-mL polypropylene tubes, with 0.025 g of activated carbon and 50 g of spiked leachate. These were shaken at room temperature and removal was measured at 1-, 5-, 10-, 20-, 40-, 80-, and 160-min intervals. At each interval, a sample of ca 5 mL was taken for analysis of organic compounds, and 0.250 mL for metal analysis. These were collected with a plastic syringe and filtered through a 0.45-μm filter. The timepoint *t* = 0 was sampled and filtered at the same time as each batch of kinetics tests were initiated.

The samples for organic analysis were weighed and isotopically labeled internal standards were added. The pH of initial and last timepoint was recorded in filtered samples with a PHM290 pH-meter (Meter lab). Samples were stored prior to organic and metal analyses at − 18 °C. The possible adsorption of analytes on the test tube walls was studied by shaking the spiked water in the tubes for 160 min. Lab blanks (consisting distilled water) were shaken for 160 min.

The metal samples were diluted to 200 times with 2% HNO_3_ solution and run directly on Agilent Triple Quadrupole inductively coupled plasma-mass spectrometer using single quadrupole mode. The organic compounds were run with a Thermo Quantiva mass spectrometer equipped with an on-line solid phase extraction column and a Hypersil Gold (50 × 2.1 mm, 3 μm particles size) analytic column (Thermo Scientific) and a HESI (heated electrospray ionization). The limit of quantification for all analytes are shown in Table S1 (Supporting information).

The removal efficiency (%) was calculated according to:

1 $$ \mathrm{removal}\ \mathrm{efficiency}\ \left(\%\right)=\left(\frac{C_i-{C}_e}{C_i}\right)\times 100\% $$

where *C*_*i*_ and *C*_*e*_ (μl L^−1^ and ng L^−1^) are the initial concentration in water and the equilibrium concentration (at 160 min), respectively. The calculation of kinetic parameters is further described in the Supporting Information.

Finally, correlations between chemical composition data obtained by XPS and adsorption efficiency were studied by linear regression analysis (by using sum of least squares method). Models with *p* < 0.05 were considered significant.

## Results and discussion

### Chemical activation of the hydrochar

**Fig. 1:**
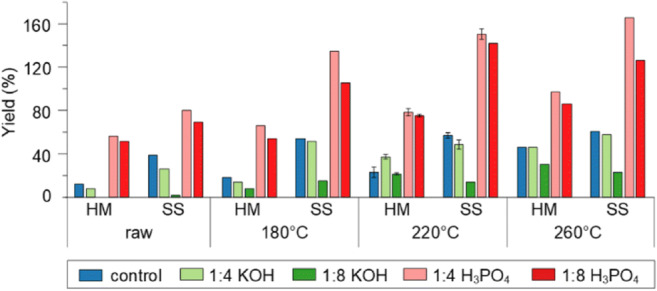
Yields of all studied activated carbons prepared from hydrochar precursors prepared at different temperatures and non-carbonized materials

Figure 1 displays the yields that were achieved after the activation of SS and HM hydrochar produced at three different HTC temperatures (180, 220, and 260 °C). In general, the activated carbon yield increased with hydrothermal temperature for both HM and SS. This is due to the differences in oxygen/carbon content of the hydrothermal precursors. As HTC temperature increases, oxygen is lost while carbon content increases, resulting in less oxygen being removed in the activation step (i.e., less mass loss).

H_3_PO_4_ activation resulted in higher yields than KOH, with some sewage sludge carbons exceeding 100%. This is attributed to the incorporation of phosphate (PO_4_^3−^) groups into the activated carbon, which can be seen in the XPS data (discussed later, “Influence of inorganic material on activation” section, Fig. 5). Since the samples were thoroughly washed after activation, the retention of phosphorus suggested that it is chemically bound into the material after activation and not loosely adsorbed to the surface. The retention of phosphorus was higher in the SS samples, potentially due to the increased level of bonding interactions between phosphate groups and the larger amount of inorganic content in SS. Retention of phosphates in the material in form of phosphate esters and condensed phosphates (Castro et al. 2000; Liou 2010; Puziy et al. 2008) is also possible. Understanding how phosphorus has bound into these materials is unfortunately beyond the scope of this study, since XANES-based studies are required for a complete understanding (Huang and Tang 2016). However, the increased yield after H_3_PO_4_ activation suggests that it may be an effective way to introduce phosphorus groups into these materials for applications that benefit from P functionalities.

### Morphology, surface area, and porosity development after chemical activation

The SS series exhibited surface areas between 34 and 343 m^2^g^−1^), which is higher than the 109 m^2^ g^−1^ reported by Kirschhofer et al. (2016) on KOH-activated hydrochar from sewage sludge. The range of surface areas here also sits in the typical range for activated sewage sludge without HTC pretreatment (6–700 m^2^ g^−1^)(Alvarez et al. 2016; Méndez et al. 2005; Ros et al. 2006; Silva et al. 2016; Wang et al. 2011). Substantially higher surface areas (< 1900 m^2^ g^−1^) have been achieved by Lillo-Ródenas et al. (2008) for sewage sludge; however, this was achieved through a series of drying, pre-activation pyrolysis at 700 °C, grinding, sieving, and then activation, a series of steps far more complex and energy consuming than HTC followed by activation.

**Fig. 2:**
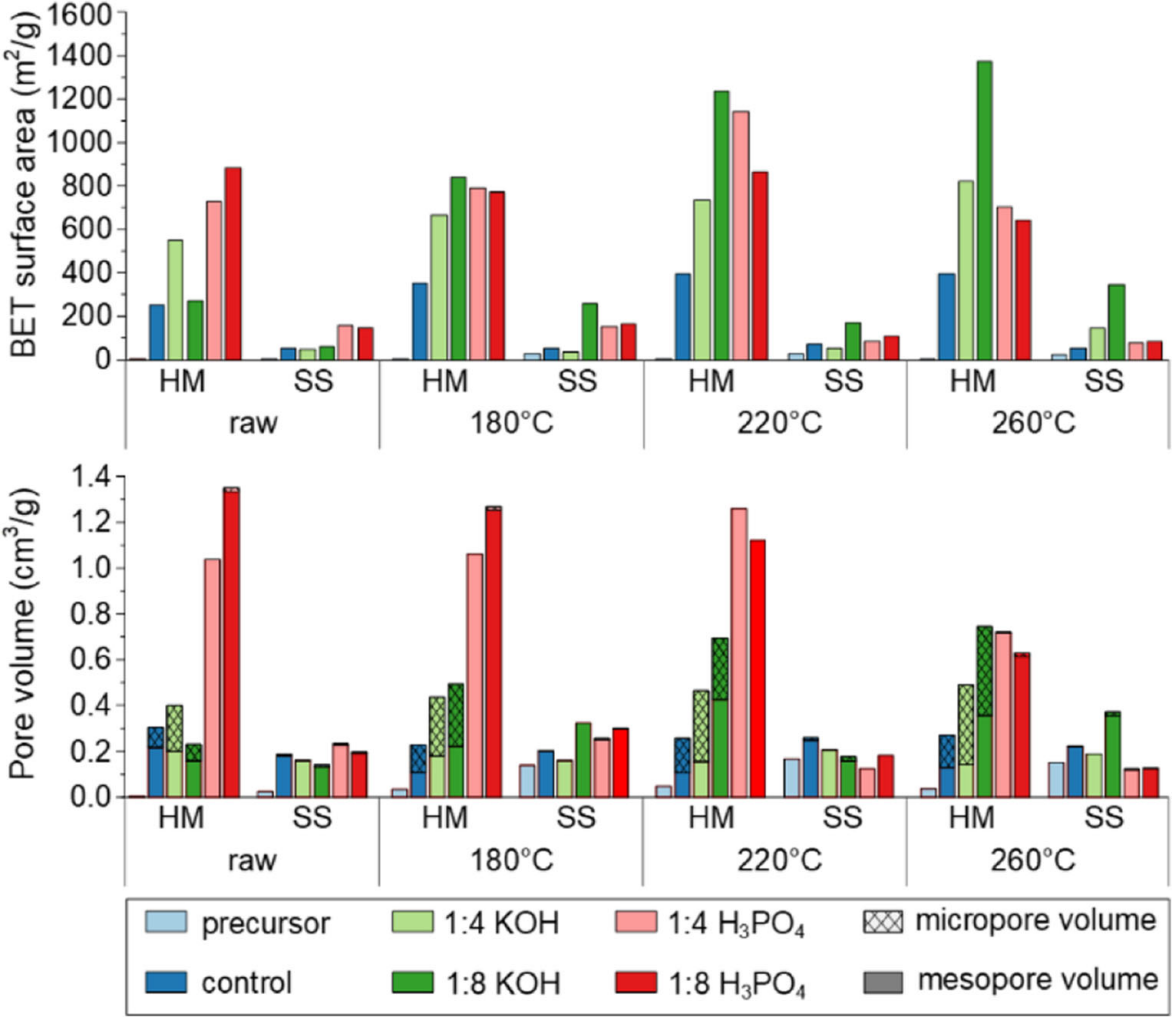
BET surface area (top) and pore volume (bottom)

The HM series produced surface areas between 230 and 1450 m^2^ g^−1^, which is higher than the SS series (34–343 m^2^ g^−1^) and a previous study examining the HTC and activation of horse manure (749 m^2^ g^−1^) (Hao et al. 2013). The higher surface area for HM, compared with SS, is thought to stem from the higher amount of inorganic content in SS that inhibits surface area development (Fig. 2). The influence of inorganic materials with regard to surface area will be discussed in the “Influence of inorganic material on activation” section with relation to the chemical characterization data.

The differences in porosity and surface area from the hydrochars activated with KOH and H_3_PO_4_ can be explained by the different activation mechanisms for KOH and H_3_PO_4_. KOH activation works through three main mechanisms: (i) etching of the carbon framework from the redox reactions between potassium compounds and carbon, (ii) gasification of the carbon from the formation of H_2_O and CO_2_, and (iii) intercalation of K into the carbon lattice followed by post-activation removal (Wang and Kaskel 2012). Alternatively, H_3_PO_4_ activation occurs through two main processes: (i) acid hydrolysis of biopolymer linkages releasing CO, CO_2_, and CH_4_, and (ii) phosphorylation of the carbon structure through the formation of phosphate esters at low temperature followed by dephosphorylation at higher temperatures and post-treatment leaching (Jagtoyen and Derbyshire 1998). The incorporation of PO_4_^−3^ units, which are larger than K^+^ ions, into the carbon structure leads to a higher level of mesopores from H_3_PO_4_ activation than KOH. However, the phosphorylation reactions needed to increase the porosity in H_3_PO_4_ activation are dependent on the biopolymer structure allowing incorporation, while KOH activation attacks the carbon structure directly to create porosity. This can be observed here with the surface area of H_3_PO_4_-activated materials decreasing at HTC temperature of 260 °C as higher HTC temperatures result in a larger aromatic/furan structures being created making it harder for phosphorylation.

**Fig. 3:**
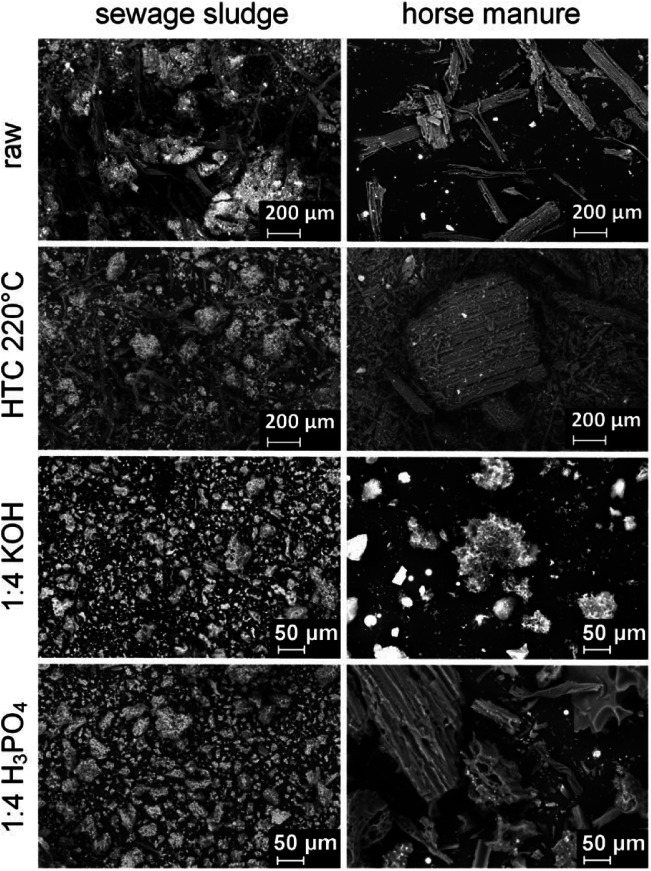
SEM pictures with × 500 magnification

The morphology of the activated carbons, observed under the SEM, was also dependent on the precursor and activation agent (Fig. 3). The SS series exhibited a range of random particles with no discernable structure that shrank after activation. HM displayed a mixture of cellulosic plant-like structures that were maintained under H_3_PO_4_, but lost after KOH activation. The morphology observed for SS and HM was maintained when the activation agent was increased form 1:4 to 1:8, although the size of the particles was reduced (Fig. S2).

### Changes in surface chemistry

The surface of HTC-activated carbon has been shown to contain a range of carbon-oxygen functionalities, such as hydroxyls, ketones/aldehydes, and carboxyls (Latham et al. 2018). These groups can participate in electrostatic and hydrogen bonding interactions increasing adsorption capacity or affinity towards certain pollutants. Additionally, the inorganic content in SS and HM also has the potential to contribute to bonding interactions. Thus, to draw correlations between the adsorption of the activated carbons, a subset was selected for in-depth FTIR and XPS analysis, followed by adsorption testing.

**Fig. 4:**
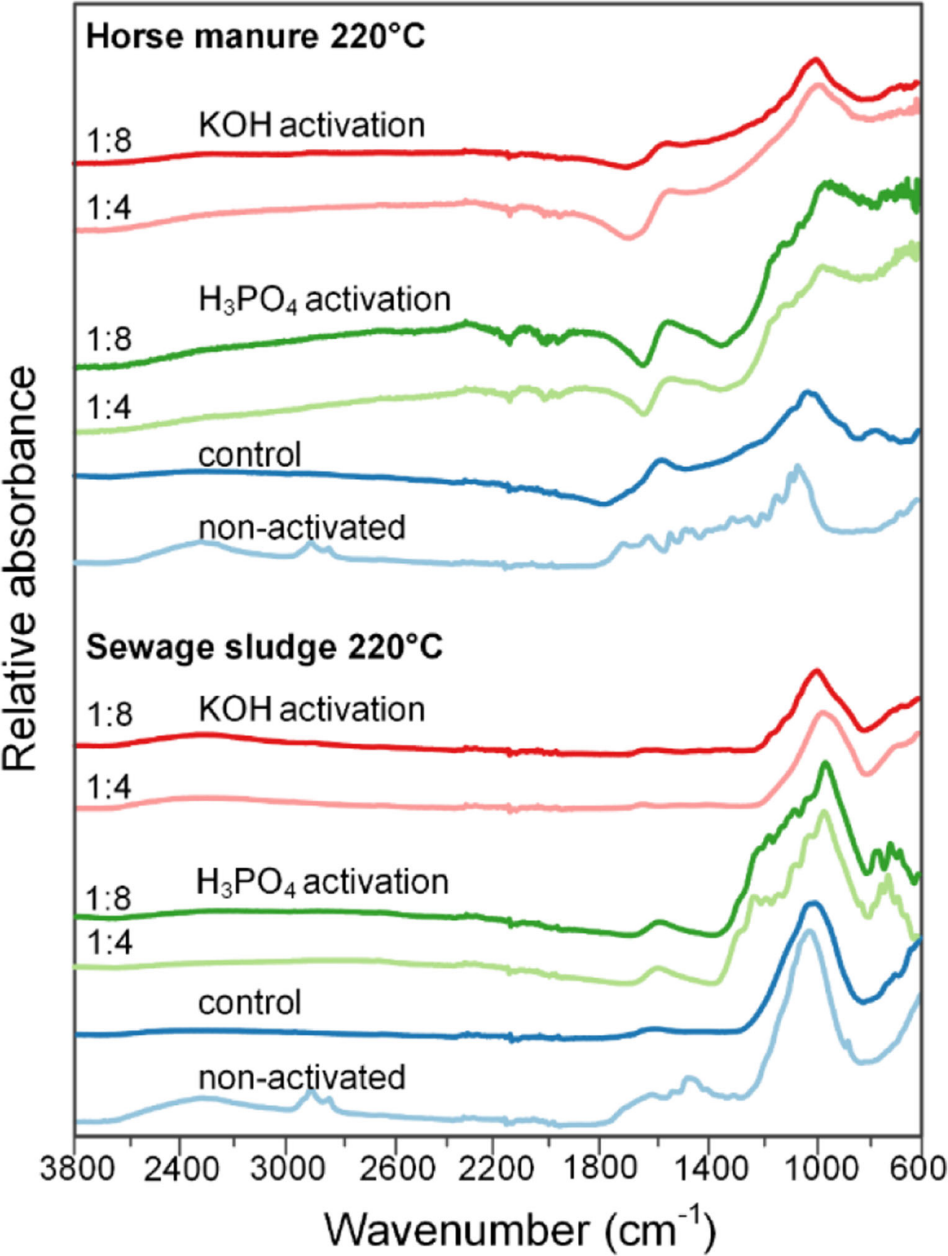
FTIR spectra of activated carbons and hydrochars used as precursors

The impact of each modification to the raw material (HTC, heat treatment, or activation) is easily observed in the FTIR spectra of HM and SS (Fig. 4). Both raw materials display a wide range of functional groups (–OH, C–O–C, C=O) intermixed with alkane/alkene and aromatic structures. After HTC treatment, some of the oxygen functionalities were removed, such as the peak for C=O at 1637 cm^−1^ in SS; however, the largest difference was observed after activation. KOH activation produced the largest alteration, with a large peak at 1350 cm^−1^ associated with mineral content and a smaller peak at 1600–1500 cm^−1^ for aromatic structures. H_3_PO_4_ activation also produced a peak at 1600–1500 cm^−1^, as well as several peaks between 1300 and 900 cm^−1^. These peaks can partly be assigned to inorganic material, but are also characteristic for phosphoric acid–activated materials that contain compounds such as phosphates and phosphoric acid esters (Liou 2010; Puziy et al. 2002). For instance, bands at ~ 1160 cm^−1^ and 1060 cm^−1^ have been assigned to C–O–P and P–O–P vibrations (Liou 2010). However, due to the large peaks associated with minerals and the fact that various C–O vibrations also occur in this region, conclusive information on the nature of P on the surface was not possible with FTIR. However, the P 2p_3/2_ peak in the XPS was located at 133.5 eV for each sample and indicated that P was in the form of PO_4_^3−^ groups bound to the surface as either phosphate esters or with inorganic materials (e.g., FePO_4_). Furthermore, the presence of H_3_PO_4_ was not detected in the XPS (134.5 eV), indicating that it had fully reacted with the hydrochars.

### Influence of inorganic material on activation

**Fig. 5:**
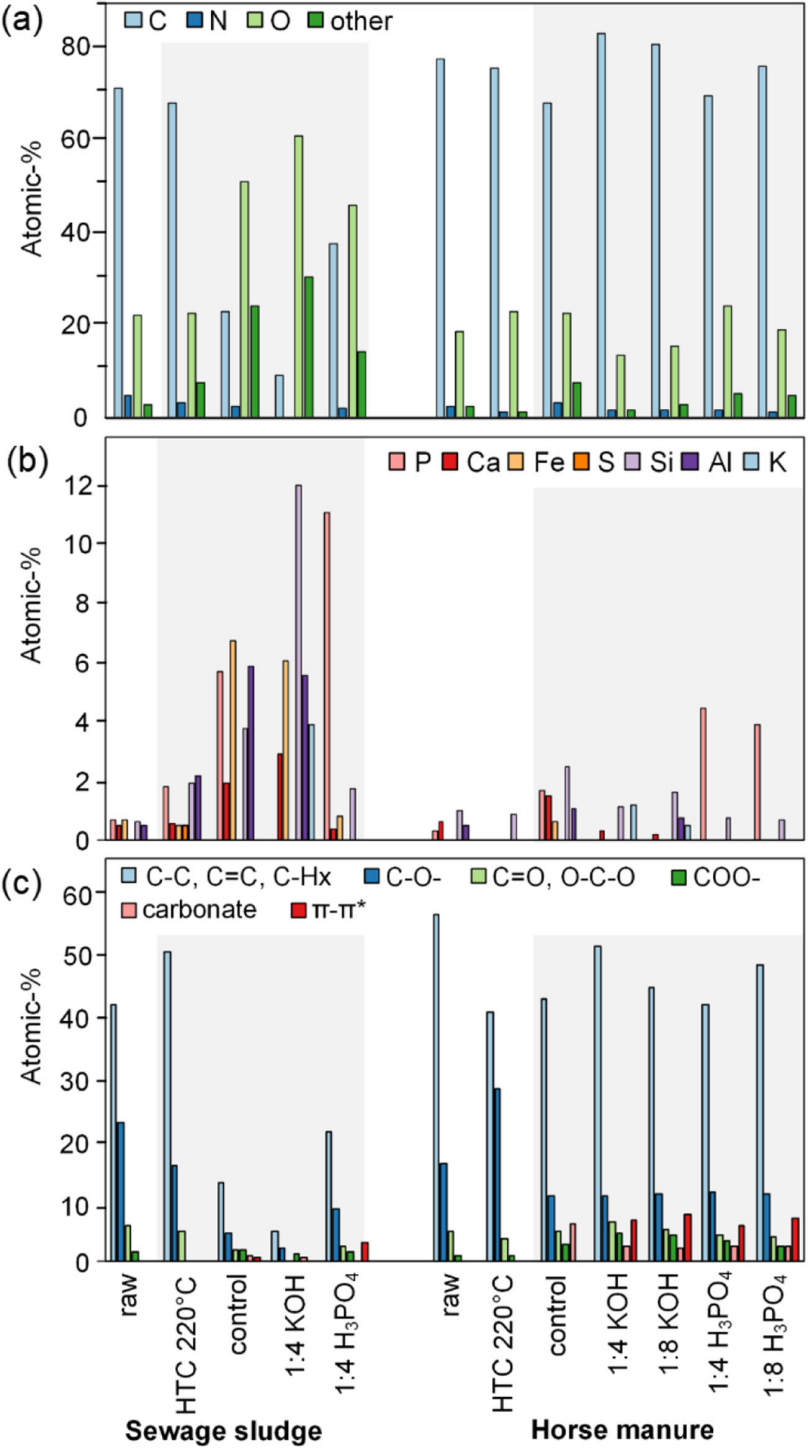
Surface elemental composition from XPS. **a** Overall carbon, oxygen, nitrogen, and other (inorganic), **b** inorganic content, and **c** deconvolution of carbon-oxygen functionalities from C1s

The inorganic material in SS appears to play a major role in determining the final composition after activation. This is less evident in the HM samples, with the material following the typical pathway for activated lignocellulosic materials, e.g., increased carbon content, surface area, and loss of oxygen. Conversely, the oxygen content increased in the SS samples after activation and heat treatment (control) (Fig. 5a). Deconvolution of the C1s peak for the SS samples indicated that this was not from the formation of carbon-oxygen groups, but from the concentration of inorganic species in the material. Carbon content was also substantially removed after heat treatment (47.88 atomic percentage (at%) loss) and activation (61.91 at% loss for KOH and 33.46 at% loss for H_3_PO_4_). This suggested an interaction between the inorganic content and the carbon structure under heat treatment (control) and activation, as the same result was not observed in HM series.

Raw SS contains a range of phosphorus, calcium, iron, silicon, and aluminum compounds, some of which are concentrated after hydrothermal treatment depending on solubility. Heat treatment (24.00 at% inorganic) and KOH activation (30.39 at% inorganic) further concentrated these compounds, while H_3_PO_4_ activation (13.92 at%) removed those which were able to be attacked by H_3_PO_4_ (Fe, Al, and Ca). Interestingly, the degree of inorganic content matched the changes in carbon content, with KOH activation having the lowest carbon content (8.89 at%) and H_3_PO_4_ activation the highest (37.34 at%), with the heat-treated sample falling in the middle (22.92 at%). This suggests that the inorganic compounds that were attacked by H_3_PO_4_ potentially play a role in removing carbon from the structure. The exact mechanism occurring here is unclear as ferrous oxides and CaCO_3_ thermally decompose at temperatures greater than 1200 °C (Qu et al. 2014), and 635 °C (Halikia et al. 2001), respectively. It is possible for some forms of aluminosilicates to decompose between 100 and 600 °C (Mayoral et al. 2001). Unfortunately, determining which component is responsible for removing the carbon is beyond the scope of this study and is better suited to a separate in-depth analysis examining these materials at multiple temperature points.

### Adsorption of contaminants

To examine how surface functionality and inorganic content influenced adsorption, a range of environmentally relevant contaminates with an assortment of functional groups and potential bonding sites were selected. These were (i) trimethoprim, which has aromatic, nitrogen and oxygen functionalities, (ii) fluconazole, which has fluorine and ring nitrogen groups, and (iii) PFOA, a fluorocarbon with a sulfur group. Additionally, copper (Cu^2+^), zinc (Zn^2+^), and arsenic (As^5+^) were selected due to environmental relevance and different possible oxidation states/ionic sizes.

**Fig. 6:**
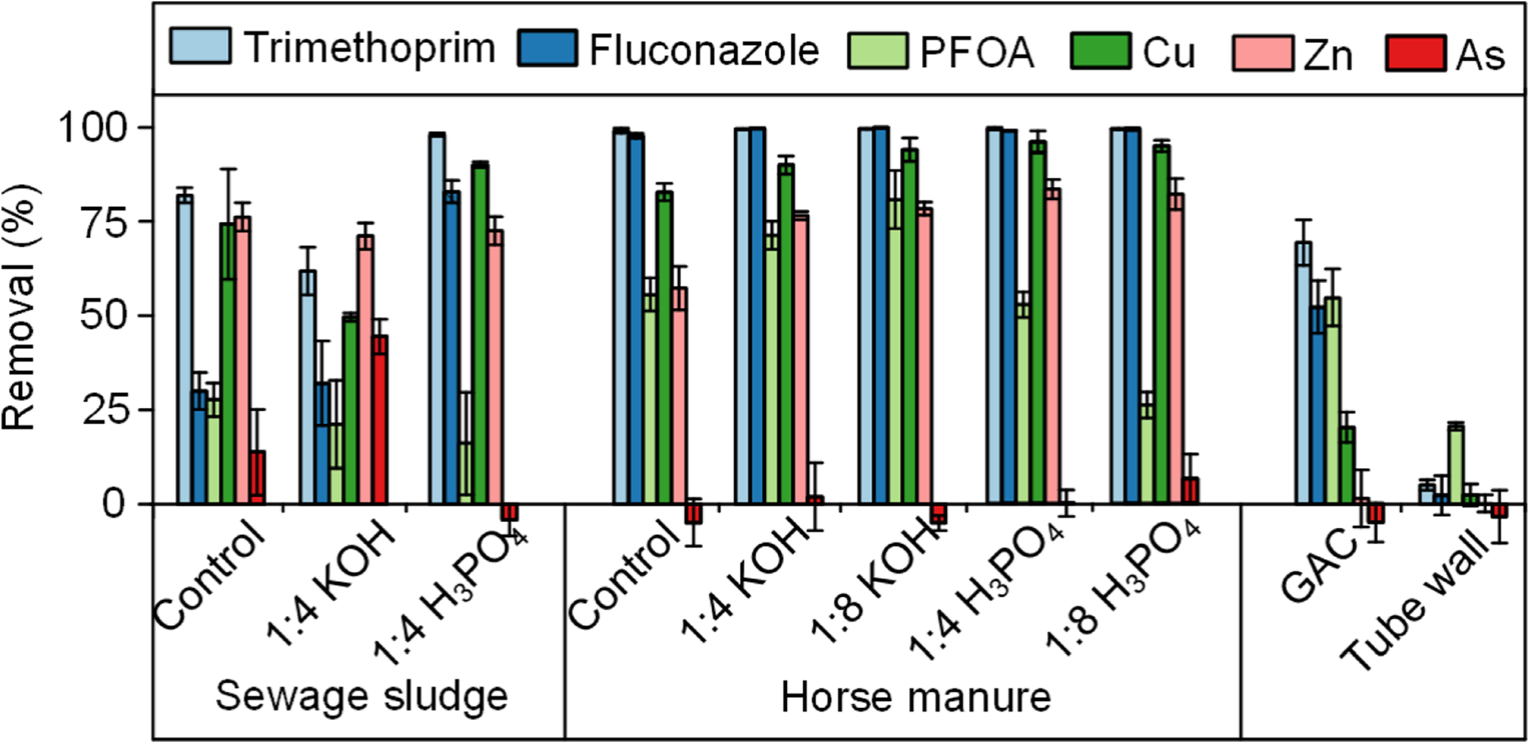
Removal efficiencies (average of triplicates at 160 min) of the studied carbon materials. The error bars show ± one standard deviation

The HM-activated carbons had a higher removal efficiency compared with SS on all contaminates other than As. The HM-activated carbons also outperformed commercial granular activated carbon (GAC), with GAC achieving only 69% trimethoprim and 52% fluconazole removal compared with > 99% from HM (Fig. 6). The activation of HM was clearly beneficial, as trimethoprim and fluconazole removal efficiencies by horse manure hydrochar without activation were ~ 25% (Weidemann et al. 2018). Despite the lower surface areas compared with HM, the SS and control samples also provided decent adsorption that outperformed the GAC sample in adsorbing several of the contaminates. For SS, the highest performance came from the H_3_PO_4_-activated carbons, which is most likely due to the higher surface area of the H_3_PO_4_ sample. Overall, the higher performance of the HM, SS, and control samples over the commercial GAC sample indicated that all of these materials are potentially viable for replacing activated carbon in wastewater treatment facilities.

**Fig. 7:**
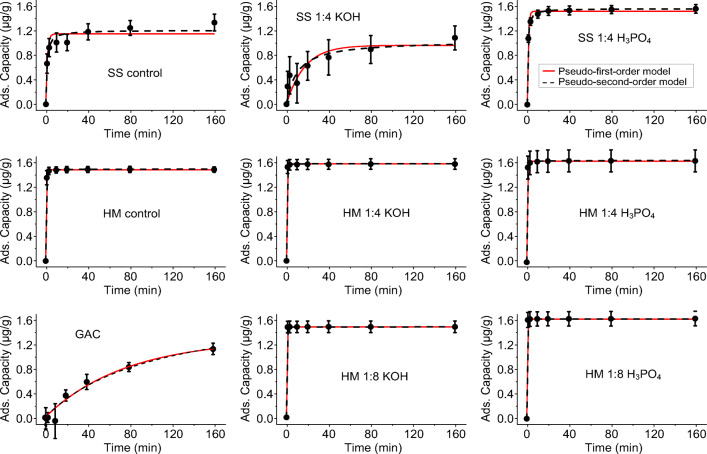
Trimethoprim removal kinetics with the studied materials. HM, SS, and GAC refers to as horse manure, sewage sludge, and granular activated carbon

Figure 7 displays the adsorption kinetics for trimethoprim, with similar adsorption shapes observed for each of the other contaminates (Supplementary Information Fig. S4-S8, Table S2). Adsorption equilibrium was reached before 160 min for most of the studied systems, while GAC showed substantially slower kinetics, which may partly explain the lower adsorption reported in this study. For the metals, SS and HM again outperformed the commercial GAC, although their maximum adsorption was less than the pharmaceuticals, indicative of the lower number of adsorption sites for metals compared with organic compounds on activated carbon.

### Effect of surface characteristics on adsorption

**Fig. 8:**
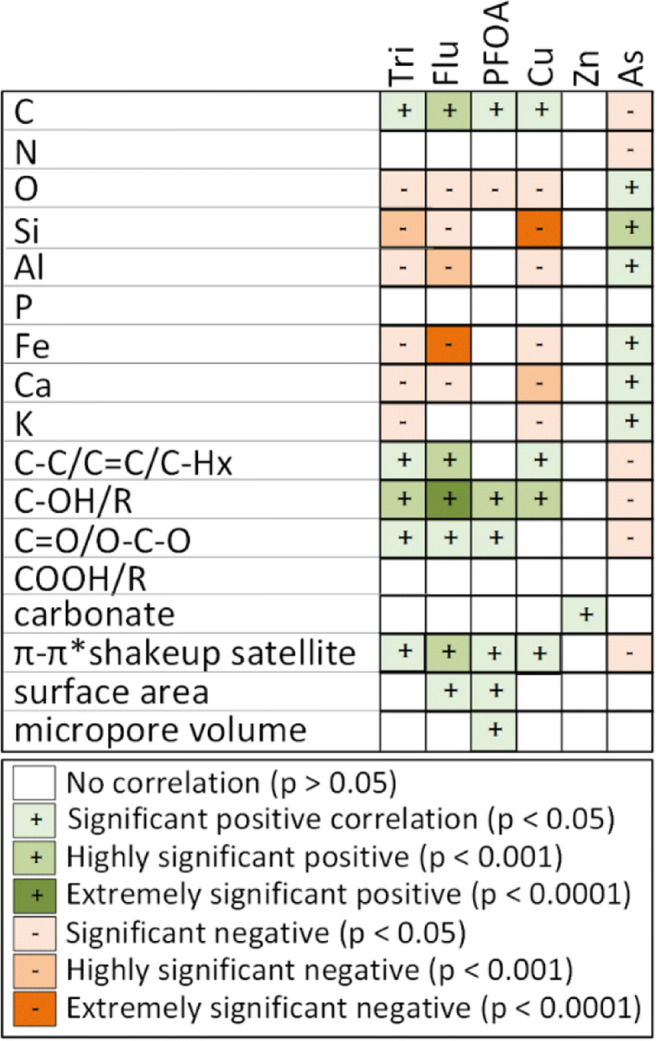
Matrix showing linearly correlated material properties and removal efficiencies of the studied contaminants. Tri, trimethoprim; Flu, fluconazole

The effect of surface properties on adsorption was studied by linear regression analysis with several material properties correlated with the removal of the studied contaminants (Fig. 8). These properties were the concentration of different organic/inorganic elements on the surface, carbon-oxygen functional groups, surface area, and micropore volume. The *R*^2^ and *p* values from the analysis are listed in the Supplementary information Table S3 for reference.

Total carbon content, aromatic/graphitic/aliphatic carbon (i.e., sp^2^ and sp^3^ carbon), oxygen functionalities (OH, C=O, and O–C–O), and π-π* shake-up satellite showed positive correlation with removal of trimethoprim, fluconazole, PFOA, and Cu, while total oxygen content and inorganic metals were negatively correlated. On the other hand, removal of As was negatively correlated with carbon and oxygen functionalities, and positively correlated with total oxygen and inorganic metals. This indicated that As is attracted to oxygenated inorganic species rather than the carbon surface or carbon-oxygen functional groups. Zn removal did not show as many correlations, which may be due to the small variation in Zn removals in the data (removal efficiencies varied only between 57 and 83%).

The high adsorption capacity of activated carbon is well known to be linked with its high surface area; however, only fluconazole and PFOA displayed correlation with surface area parameter. This is likely due to using a linear model for analysis instead of an exponential model. When comparing the surface area vs removal efficiency for trimethoprim, fluconazole, and Cu, an exponential relationship can be easily observed (Fig. S9), suggesting that surface area does have a positive correlation with removal efficiency.

These findings suggest that surface oxygen functionalities may have an important role in removal, as possible binding sites for charged and polar contaminants by hydrogen bonds and electrostatic interactions (Fang et al. 2014; Shi et al. 2015). On the other hand, sp^2^ and sp^3^ carbon provide sites for physisorption or π-π interactions (Jung et al. 2013; Sun et al. 2012). Removal of As may be governed by precipitation, which has been previously shown to occur with iron (Escudero et al. 2009; Roberts et al. 2004) or interactions with metal oxides. It should be noted though that this data does not distinguish between causation and co-variation. For instance, sp^2^/sp^3^ carbon content has very strong positive correlation with π-π* shake-up satellite and total carbon content (correlations between all material features are shown in Table S4), meaning that correlation with one of these factors will very likely induce correlation with other co-varying factors. However, the correlations have shown that inorganic components may negatively impact the ability for these materials to remove trimethoprim, fluconazole, and Cu, while carbon-oxygen groups provide bonding sites. This suggests that it is beneficial to remove the inorganic components during the HTC process before activation.

## Conclusions

This study examined the activation of hydrothermally carbonized horse manure and sewage sludge. The hydrochars produced at higher temperatures had higher surface areas and yield, indicating a link between hydrothermal temperature and activation. Chemical activation with KOH produced higher surface area, while H_3_PO_4_ had higher yields. Additionally, the hydrochars activated with H_3_PO_4_ had substantially less inorganic content than their KOH counterparts. The adsorption studies were performed in a real-world matrix, with the materials able to remove nearly all traces of trimethoprim, fluconazole, Cu, and Zn, while PFOA and As removal was low. Linear regression analysis showed strong positive correlations between carbon-oxygen surface functionalities and the removal of trimethoprim, fluconazole, PFOA, and Cu. On the other hand, As displayed a completely opposite trend. This suggested that different removal mechanisms are required to remove various different contaminates and the need to design versatile adsorbent materials.

## Electronic supplementary material

ESM 1 (DOCX 2192 kb)
