# Supplementary material for: The influence of inorganic components and carbon-oxygen surface functionalities in activated hydrothermally carbonized waste materials for water treatment
Source: Environ Sci Pollut Res Int. 2020 Jul 3;27(30):38072–83. doi: 10.1007/s11356-020-09839-1 (PMC7496029; doi:10.1007/s11356-020-09839-1)
Supplement: Supplementary file 1 — (DOCX 2192 kb) [file 11356_2020_9839_MOESM1_ESM.docx]

**SUPPORTING INFORMATION**

The Influence of Inorganic Components and Carbon-Oxygen Surface Functionalities for Water Treatment in Activated Hydrothermally Carbonized Waste Materials

Mirva Niinipuu, ^†,‡^ Kenneth G. Latham, ^†^ Stina Jansson ^† *^

^†^ Department of Chemistry, Umeå University, SE-90187, Umeå, Sweden

^‡^ Industrial Doctoral School, Umeå University, SE-90187, Umeå, Sweden

* Corresponding author: stina.jansson@umu.se

**
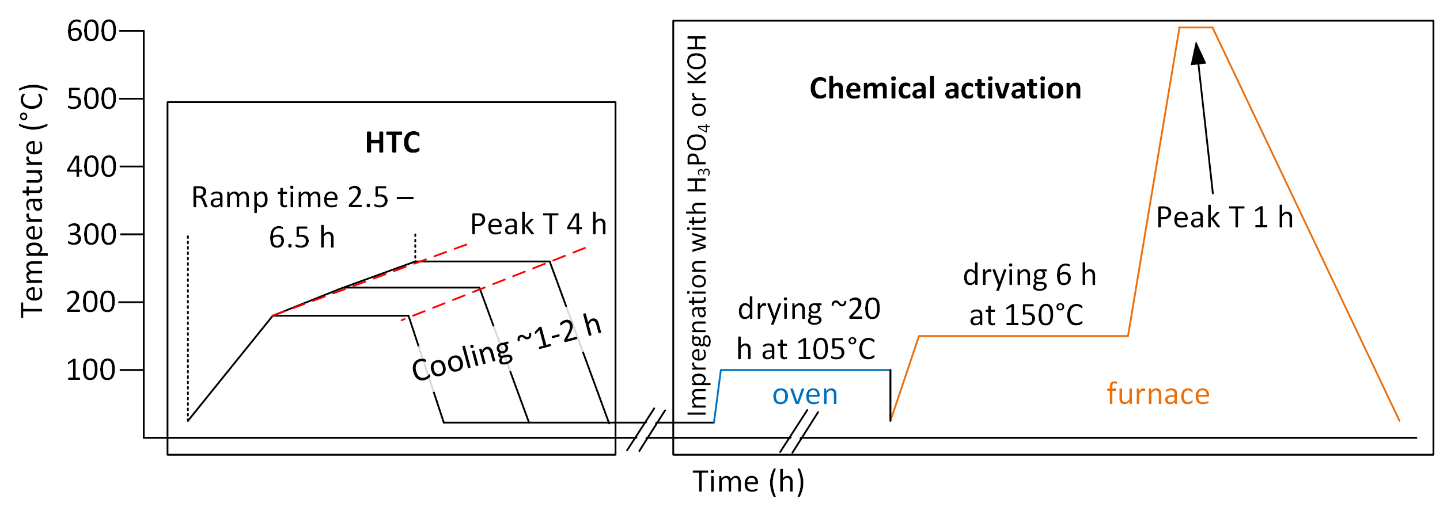
**

**Figure S1.** Temperature ramps of HTC and chemical activation.

**Table S1**. Limit of quantification (LOQ) for the analytes.

| Analyte | Unit | LOQ | Method |  |
| --- | --- | --- | --- | --- |
| Cu | µg/l | 0.1 | ICP-MS |  |
| Zn | µg/l | 0.3 | ICP-MS |  |
| As | µg/l | 0.02 | ICP-MS |  |
| Fluconazole | ng/l | 1 | LC-MS |  |
| Trimethoprim | ng/l | 5 | LC-MS |  |
| PFOA | ng/l | 30 | LC-MS |  |

**Calculation of removal kinetics**

The removal kinetics were further studied by fitting the data into non-linear pseudo-first- and second order models. Non-linear Lagergren pseudo-first order model (PFO)^1^ is expressed as:

$q_{t}=q_{e}(1-e^{-k_{1}t})$ (1)

Where *q_t_* (ng g^-1^ or µg g^-1^) is the adsorption capacity at time = *t* and *q_e_* (ng g^-1^ or µg g^-1^) is the adsorption capacity at equilibrium and k_1_ (min^-1^) is the pseudo-first order rate constant. Adsorption capacity, *q_t_* (μl L^-1^ and ng L^-1^), at time point *t* (min) was calculated as follows:

$q_{t}=\frac{(C_{i}-C_{t})}{m}V$ (2)

Where *C_t_* (μl L^-1^ and ng L^-1^) is the concentration in water at the time point *t*, *m* (g) is the mass of the adsorbent, and *V* (L) is the water volume.

The non-linear form of the pseudo-second order model (PSO) ^2^ was also fitted to the obtained data and it is expressed as:

$q_{t}=\frac{q_{e}^{2}k_{2}t}{1+q_{e}k_{2}t}$ (3)

Where *k_2_* (g ng^-1^ min^-1^ or g µg^-1^ min^-1^) is the pseudo-first order rate constant.


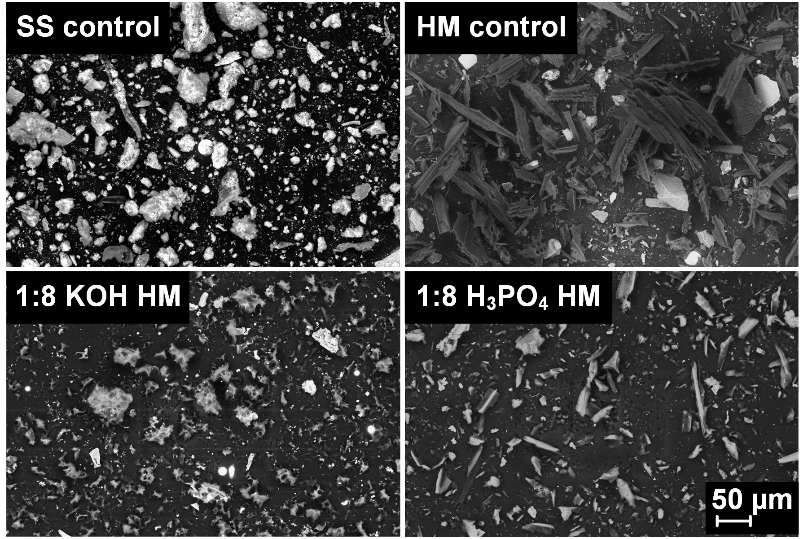


**Figure S2.** SEM images of control samples and 1:8 activated samples.


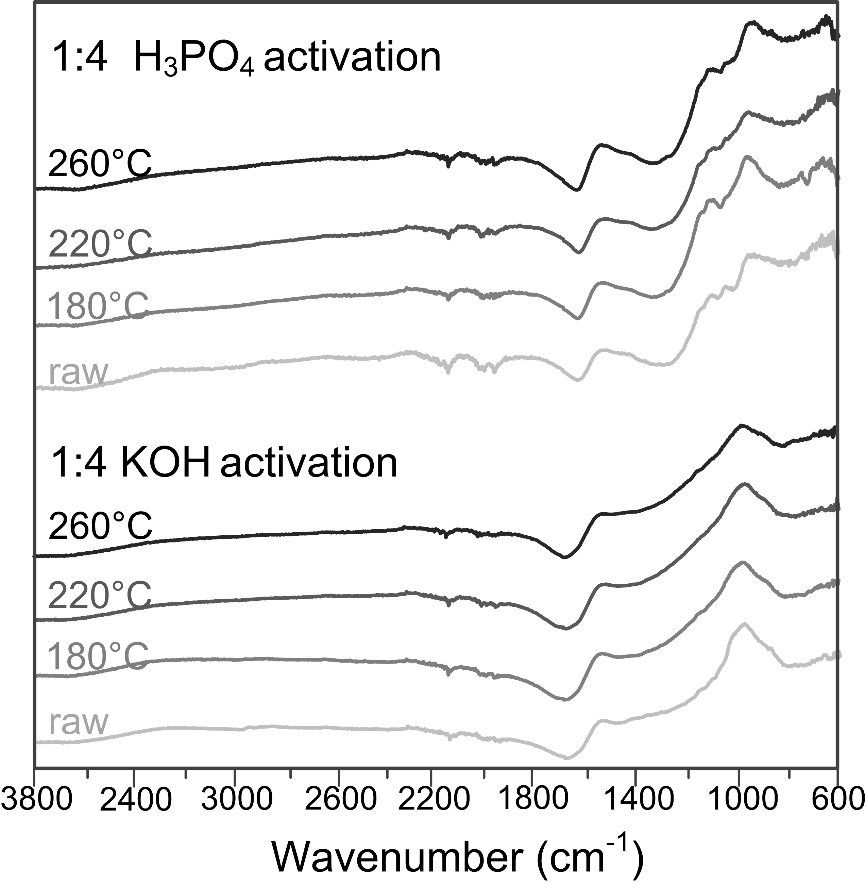


**Figure S3.** Chemically activated temperature series of horse manure hydrochars.

**Figure S4.** Fluconazole removal kinetics with the studied materials. HM, SS and GAC refers to as horse manure, sewage sludge and granular activated carbon, respectively.

**Figure S5.** PFOA removal kinetics with the studied materials. HM, SS and GAC refers to as horse manure, sewage sludge and granular activated carbon, respectively.

**Figure S6.** Cu removal kinetics with the studied materials. HM, SS and GAC refers to as horse manure, sewage sludge and granular activated carbon, respectively.

**Figure S7.** Zn removal kinetics with the studied materials. HM, SS and GAC refers to as horse manure, sewage sludge and granular activated carbon, respectively.

**Figure S8.** As removal kinetics for materials removing As. HM and SS refers to as horse manure and sewage sludge, respectively.

**Table S2.** Pseudo first order (PFO) and pseudo-second order model (PSO) parameters, q_e_ (μg/g), k_1_ (min^-1^), k_2_ (g μg^-1^ min^-1^) and R^2^.

|  | | **PFO** | | | **PSO** | | |  |  | **PFO** | | | **PSO** | | |
| --- | --- | --- | --- | --- | --- | --- | --- | --- | --- | --- | --- | --- | --- | --- | --- |
| **Trimethoprim** | | q_e_ | k_1_ | R^2^ | q_e_ | k_2_ | R^2^ |  | **Arsenic** | q_e_ | k_1_ | R^2^ | q_e_ | k_2_ | R^2^ |
| Horse  manure | control | 1.488 | 2.438 | 1.000 | 1.499 | 6.757 | 1.000 |  |  | - | - | - | - | - | - |
|  | 1:4 KOH | 1.576 | 3.595 | 1.000 | 1.580 | 21.27 | 1.000 |  |  | - | - | - | - | - | - |
|  | 1:8 KOH | 1.478 | 5.827 | 1.000 | 1.478 | 207.3 | 1.000 |  |  | - | - | - | - | - | - |
|  | 1:4 H_3_PO_4_ | 1.647 | 2.791 | 1.000 | 1.657 | 8.514 | 1.000 |  |  | - | - | - | - | - | - |
|  | 1:8 H_3_PO_4_ | 1.630 | 4.470 | 1.000 | 1.632 | 49.20 | 1.000 |  |  | - | - | - | - | - | - |
| Sewage sludge | control | 1.146 | 0.735 | 0.910 | 1.205 | 0.882 | 0.952 |  |  | 8.93 | 0.045 | 0.591 | 9.83 | 0.00668 | 0.564 |
|  | 1:4 KOH | 0.957 | 0.058 | 0.742 | 1.034 | 0.093 | 0.809 |  |  | 19.5 | 0.030 | 0.864 | 23.4 | 0.00141 | 0.870 |
|  | 1:4 H_3_PO_4_ | 1.514 | 1.173 | 0.988 | 1.559 | 1.430 | 1.000 |  |  | - | - | - | - | - | - |
| GAC |  | 1.284 | 0.013 | 0.954 | 1.894 | 0.005 | 0.951 |  |  | - | - | - | - | - | - |
|  |  |  |  |  |  |  |  |  |  |  |  |  |  |  |  |
| **Fluconazole** | |  |  |  |  |  |  |  | **Copper** |  |  |  |  |  |  |
| Horse  manure | control | 1.363 | 0.928 | 0.980 | 1.411 | 1.135 | 0.999 |  |  | 682 | 0.770 | 0.940 | 713 | 0.00167 | 0.980 |
|  | 1:4 KOH | 1.531 | 1.278 | 0.990 | 1.573 | 1.617 | 1.000 |  |  | 784 | 0.760 | 0.885 | 820 | 0.00144 | 0.950 |
|  | 1:8 KOH | 1.414 | 3.744 | 1.000 | 1.418 | 25.67 | 1.000 |  |  | 779 | 1.791 | 0.984 | 795 | 0.00532 | 0.993 |
|  | 1:4 H_3_PO_4_ | 1.489 | 1.566 | 0.990 | 1.523 | 2.264 | 0.999 |  |  | 884 | 1.216 | 0.965 | 913 | 0.00240 | 0.986 |
|  | 1:8 H_3_PO_4_ | 1.492 | 2.559 | 0.999 | 1.504 | 7.124 | 1.000 |  |  | 897 | 1.656 | 0.991 | 916 | 0.00422 | 0.998 |
| Sewage sludge | control | 0.395 | 1.026 | 0.735 | 0.414 | 3.678 | 0.771 |  |  | 628 | 0.143 | 0.791 | 707 | 0.00024 | 0.829 |
|  | 1:4 KOH | 0.479 | 0.038 | 0.464 | 0.498 | 0.152 | 0.539 |  |  | 397 | 0.062 | 0.724 | 411 | 0.00032 | 0.810 |
|  | 1:4 H_3_PO_4_ | 1.102 | 0.510 | 0.909 | 1.167 | 0.622 | 0.967 |  |  | 789 | 0.655 | 0.940 | 828 | 0.00118 | 0.977 |
| GAC |  | 1.013 | 0.010 | 0.938 | 1.572 | 0.004 | 0.936 |  |  | 362 | 1.016 | 0.422 | 380 | 0.00402 | 0.463 |
|  |  |  |  |  |  |  |  |  |  |  |  |  |  |  |  |
| **PFOA** |  |  |  |  |  |  |  |  | **Zink** |  |  |  |  |  |  |
| Horse  manure | control | 0.914 | 2.732 | 0.912 | 0.937 | 5.927 | 0.929 |  |  | 550 | 0.833 | 0.956 | 572 | 0.00241 | 0.990 |
|  | 1:4 KOH | 0.985 | 1.848 | 0.765 | 1.028 | 2.628 | 0.801 |  |  | 820 | 0.832 | 0.985 | 851 | 0.00165 | 0.997 |
|  | 1:8 KOH | 1.305 | 1.106 | 0.882 | 1.369 | 1.178 | 0.942 |  |  | 793 | 1.681 | 0.986 | 811 | 0.00469 | 0.996 |
|  | 1:4 H_3_PO_4_ | 0.761 | 2.094 | 0.795 | 0.801 | 3.149 | 0.845 |  |  | 845 | 0.247 | 0.984 | 914 | 0.00036 | 0.993 |
|  | 1:8 H_3_PO_4_ | 0.369 | 20.00 | 0.561 | 0.389 | 1.57E+34 | 0.577 |  |  | 828 | 1.376 | 0.966 | 853 | 0.00307 | 0.984 |
| Sewage sludge | control | 0.463 | 0.025 | 0.310 | 0.550 | 0.053 | 0.327 |  |  | 684 | 0.552 | 0.693 | 757 | 0.00068 | 0.799 |
|  | 1:4 KOH | 0.476 | 20.000 | 0.657 | 0.466 | 2.33E+25 | 0.639 |  |  | 617 | 0.497 | 0.894 | 658 | 0.00101 | 0.955 |
|  | 1:4 H_3_PO_4_ | 0.230 | 2.253 | 0.525 | 0.242 | 10.854 | 0.558 |  |  | 731 | 0.509 | 0.919 | 776 | 0.00090 | 0.971 |
| GAC |  | 1.128 | 0.018 | 0.795 | 1.539 | 0.010 | 0.790 |  |  | 312 | 0.113 | 0.404 | 327 | 0.00054 | 0.320 |

**Table S3.** R^2^ and p-values obtained for linear correlations. White, green and red backgrounds indicate no correlation, positive and negative correlations (*p* < 0.05), respectively.

|  | trimethoprim | | fluconazole | | PFOA | | Cu | | Zu | | As | |
| --- | --- | --- | --- | --- | --- | --- | --- | --- | --- | --- | --- | --- |
|  | R^2^ | *p* | R^2^ | *p* | R^2^ | *p* | R^2^ | *p* | R^2^ | *p* | R^2^ | *P* |
| C | 0.740 | 0.0061 | 0.866 | 0.00079 | 0.565 | 0.032 | 0.692 | 0.010 |  |  | 0.549 | 0.036 |
| N |  |  |  |  |  |  |  |  |  |  | 0.508 | 0.047 |
| O | 0.701 | 0.0095 | 0.823 | 0.0019 | 0.599 | 0.024 | 0.640 | 0.017 |  |  | 0.5120 | 0.044 |
| Si | 0.928 | 0.00012 | 0.609 | 0.022 |  |  | 0.946 | 0.000050 |  |  | 0.857 | 0.00096 |
| Al | 0.796 | 0.0023 | 0.928 | 0.00012 |  |  | 0.752 | 0.0053 |  |  | 0.613 | 0.022 |
| P |  |  |  |  |  |  |  |  |  |  |  |  |
| Fe | 0.792 | 0.0030 | 0.985 | 0.0000011 |  |  | 0.728 | 0.0071 |  |  | 0.614 | 0.021 |
| ca | 0.804 | 0.0025 | 0.730 | 0.0069 |  |  | 0.931 | 0.00011 |  |  | 0.626 | 0.019 |
| K | 0.672 | 0.013 |  |  |  |  | 0.669 | 0.013 |  |  | 0.745 | 0.0058 |
| C-C/C=C/C-Hx | 0.722 | 0.0076 | 0.858 | 0.00094 |  |  | 0.658 | 0.015 |  |  | 0.509 | 0.047 |
| C-O- | 0.919 | 0.00017 | 0.952 | 0.000035 | 0.882 | 0.00054 | 0.859 | 0.00093 |  |  | 0.759 | 0.0048 |
| C=O/O-C-O | 0.736 | 0.0065 | 0.771 | 0.0041 | 0.662 | 0.014 |  |  |  |  | 0.621 | 0.020 |
| COO- |  |  |  |  |  |  |  |  |  |  |  |  |
| carbonate |  |  |  |  |  |  |  |  | 0.637 | 0.018 |  |  |
| Pi-pi | 0.733 | 0.0066 | 0.901 | 0.00031 | 0.515 | 0.045 | 0.683 | 0.011 |  |  | 0.552 | 0.035 |
| surface area |  |  | 0.549 | 0.035 | 0.519 | 0.044 |  |  |  |  |  |  |
| micropore |  |  |  |  | 0.741 | 0.0060 |  |  |  |  |  |  |


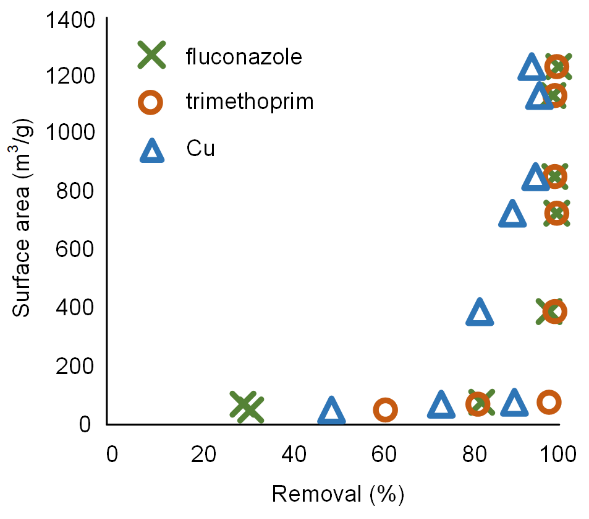


**Figure S9.** Surface area and removal of fluconazole, trimethoprim and Cu are not well described by a linear model.

**Table S4.** Matrix showing co-variation of different carbon properties and correlation *p*-values. White, green and red backgrounds indicate no correlation (*p* > 0.05), positive and negative correlations (*p* < 0.05), respectively.

|  | N | O | Si | Al | P | Fe | ca | K | C-C/  C=C/  C-Hx | C-O- | C=O/  O-C-O | COO- | carbonate | pi-pi* | SSA | Micro  pore |
| --- | --- | --- | --- | --- | --- | --- | --- | --- | --- | --- | --- | --- | --- | --- | --- | --- |
| C |  | 0.000000063 | 0.021 | 0.011 |  | 0.0030 | 0.014 |  | 0.00000069 | 0.00033 | 0.00022 | 0.0056 |  | 0.0000071 | 0.0088 |  |
| N |  |  |  |  |  |  |  | 0.032 |  |  |  |  |  |  |  |  |
| O |  |  | 0.027 | 0.019 |  | 0.0062 | 0.025 |  | 0.0000012 | 0.00086 | 0.00015 | 0.0044 |  | 0.000022 | 0.011 |  |
| Si |  |  |  | 0.021 |  | 0.027 | 0.0028 | 0.0026 | 0.023 | 0.0036 | 0.022 |  |  | 0.029 |  |  |
| Al |  |  |  |  |  | 0.0000075 | 0.0022 |  | 0.011 | 0.00092 | 0.023 |  |  | 0.0072 |  |  |
| P |  |  |  |  |  |  |  |  |  |  |  |  |  |  |  |  |
| Fe |  |  |  |  |  |  | 0.0045 |  | 0.0036 | 0.00022 | 0.010 |  |  | 0.0013 | 0.048 |  |
| Ca |  |  |  |  |  |  |  |  | 0.020 | 0.0032 | 0.039 |  |  | 0.014 | 0.036 |  |
| K |  |  |  |  |  |  |  |  |  |  |  |  |  |  |  |  |
| C-C/C=C/C-Hx | | |  |  |  |  |  |  |  | 0.00047 | 0.00034 | 0.011 |  | 0.000048 | 0.016 |  |
| C-O- |  |  |  |  |  |  |  |  |  |  | 0.0021 | 0.042 |  | 0.00031 | 0.027 |  |
| C=O/O-C-O | |  |  |  |  |  |  |  |  |  |  | 0.0028 |  | 0.0013 |  | 0.040 |
| COO- |  |  |  |  |  |  |  |  |  |  |  |  | 0.038 | 0.015 | 0.013 | 0.018 |
| carbonate | |  |  |  |  |  |  |  |  |  |  |  |  |  | 0.010 |  |
| pi-pi* |  |  |  |  |  |  |  |  |  |  |  |  |  |  | 0.0087 |  |
| SSA |  |  |  |  |  |  |  |  |  |  |  |  |  |  |  |  |

**References:**

(1) Lagergren, S. Zur Theorie Der Sogenannten Adsorption Gelöster Stoffe. *K. Sven. Vetenskapsakademiens. Handl.* **1898**, *24* (4), 1–39.

(2) Blanchard, G.; Maunaye, M.; Martin, G. Removal of Heavy Metals from Water. *Water Res.* **1984**, *18* (12), 1501–1507.
